# Child–Dog Attachment, Emotion Regulation and Psychopathology: The Mediating Role of Positive and Negative Behaviours

**DOI:** 10.3390/bs12040109

**Published:** 2022-04-15

**Authors:** Roxanne D. Hawkins, Charlotte Robinson, Zara P. Brodie

**Affiliations:** 1 Division of Psychology, School of Education and Social Sciences, Paisley Campus, University of West of Scotland, Elles Building East, Paisley PA1 2BE, UK; zara.brodie@uws.ac.uk; 2 School of Psychology, Cardiff University, Tower Building, 70 Park Place, Cardiff CF10 3AT, UK; robinsonc4@cardiff.ac.uk

**Keywords:** attachment, dogs, child development, companion animals, emotion regulation, human–animal interaction, pets, psychopathology

## Abstract

Emerging evidence suggests that pet dogs can offer features of a secure attachment which has been associated with healthy psychological development across the lifespan. Limited research has investigated the underpinning mechanisms that may contribute to the benefits and risks of child–dog attachment during childhood. This study aimed to test the potential mediating role of caregiver-observed positive and negative child–dog behaviours, on the relationship between child-reported child–dog attachment, and caregiver-reported child psychopathology and emotion regulation. Data from 117 caregiver reports and 77 child self-reports were collected through an online survey in the context of the COVID-19 pandemic. Parallel mediation analyses indicated that child–dog attachment had a significant indirect effect on conduct problems through negative child–dog behaviours only. Child–dog attachment had a significant indirect effect on emotional symptoms, peer problems, prosocial behaviour, emotion regulation, and emotional lability/negativity through both positive and negative child–dog behaviours. Although this study found modest effect sizes, the findings suggest that the types of interactions that children engage in with their pet dogs may be important mechanisms through which pet attachment contributes to psychological development throughout childhood, and therefore further attention is warranted. Positive and safe child–dog interactions can be facilitated through education and intervention, which may have implications for promoting positive developmental outcomes.

## 1. Introduction

Growing up with childhood pets is common, and such early experiences in human–animal interactions (HAIs) confer both risk and benefits to a young person’s development, depending on the type of interactions that exist between them and the strength of the human–pet bond. Many child–pet relationships are positive ones, as animals are often viewed by children as being central to their family and social systems and are often granted the status of ‘best friend’; this bond and friendship are often perceived as being reciprocal [1,2,3]. Children are often found to be emotionally expressive toward their pets and turn to them for support and comfort, particularly in times of distress and adversity, which can increase resilience and protect against psychopathology [4,5,6]. Dogs especially can serve a therapeutic function, particularly for children with emotional problems through increasing emotional stability, evidenced by the prevention and de-escalation of episodes of emotional crisis [7,8]. Children have an innate motivation to care for and engage in positive interactions with animals that could positively impact their psychological development [9,10]. The types of activities that children engage in with their pets are important and play different functions. For example, physical caretaking behaviour (e.g., dog walking) may increase children’s physical health and facilitate social contact, whereas physical touch and other affectionate behaviour may relieve stress and anxiety through decreased physiological arousal and increased hedonic mood [11,12,13]. Many of these positive caring interactions are encouraged by adult caregivers [14]. Moreover, previous research has found associations between play behaviour, (including spontaneous, pretend, and more structured play) and positive impacts on self-control and impulse control, behaviour inhibition, and adaptive skills [15,16,17]. It is therefore possible that child–dog play, a positive feature of HAIs, could foster emotion regulation in children [16]. It is also possible that speaking to a pet dog positively (rather than reprimanding and shouting), could also play a key role in self-regulation development because self-regulation mediates the relationship between language and physical aggression [18].

Derived benefits of childhood pets for development may only exist for those who spend quality time with and engage in positive interactions with their pets, are strongly attached to them, and are directly involved and responsible for their care [19,20]. Pet attachment is important for childhood development yet is often overlooked in HAI research, possibly explaining the mixed and inconclusive evidence when investigating proposed beneficial outcomes of HAIs on childhood development [10]. Human–pet relationships often meet the prerequisites of an attachment relationship, displaying similar features to human–human attachments (proximity seeking, safe-haven, secure base, separation distress) [21,22,23,24] but can also feature insecurities, anxieties, avoidance, and negative expectations [24,25,26]. The strength of a child’s pet attachment may impact the types of behaviours that children engage in with their animal, possibly impacting both the treatment of the pet and the child’s psychological development [27]. For example, a strong attachment may facilitate a nurturing reaction, leading to caregiving and protective behaviours directed toward the pet from the child [14,28,29], whereas a weaker or absent attachment may increase the risk for animal neglect and negative interactions including the acceptance of animal cruelty [30,31,32]. Child–pet attachment should therefore be considered to disentangle the potential impacts of pets on children’s developmental outcomes. Child–pet relationships are multi-layered and complex, and they are not always positive. It is not uncommon for children and young people to engage in negative and unsafe interactions with their pets even when an attachment is present, inflicting unintentional harm or neglect onto their animal, either through curious childhood play or through a lack of understanding and knowledge of appropriate and safe ways to care for and interact with their animal [28,33,34]. This poses risk for both child development and animal welfare. However, existing research has mostly focused on positive features of HAIs and has overlooked the consideration that negative interactions may be occurring, possibly increasing the risk for emotional and behavioural problems.

A paucity of research has highlighted the psychosocial and psychophysiological effects of HAIs [35,36] and has pointed to the positive impacts of childhood pets for socioemotional and behavioural development [10,37]. For example, it has been suggested that engagement with animals can help children to self-regulate, including fostering the social regulation of emotion and enhancing cognitive control through the animals’ ability to respond to the child’s attachment-related behaviour [23,38,39]. It is well-established that difficulties with self-regulation, including difficulties with emotion regulation (successful management of emotional arousal), can impact psychological adjustment, contributing to emotional and behavioural problems in childhood. Examples include an increased risk of externalizing problems and conduct disorder and a negative impact on interpersonal relationships [40,41]. Such regulation skills may also be important for negotiating complex interactions with animals, with poor self-regulation potentially posing a risk for negative and unsafe interactions with pets. Although no research to date has directly examined poor emotion regulation abilities and negative HAIs, research does point to a potential link between childhood animal cruelty and poor impulsivity and ‘acting out’, poor self-control, and displaced frustration [32,42,43]. Moreover, it is well known from human attachment research that insecure attachment increases the risk of difficulties in emotion regulation [44,45]; however, this has not yet been examined in pet attachment research. Emotion regulation may therefore impact the human–animal bond and, consequently, children’s positive engagement with pets, making it an important focus in the current investigation.

Research has indicated the beneficial effects of animals for reducing problematic behaviour in childhood [46], and such benefits could be explained through an increase in self-esteem, empathy, sense of responsibility, and social competence [47,48]. This is important because one of the most common reasons for referral to a childhood mental health service is externalizing problems, and so understanding how children can develop skills to regulate behaviour and emotions is important [49]. This will have subsequent implications for animal welfare because children and young people who have engaged in negative interactions with animals, including direct animal harm, tend to display more conduct problems, delinquency, and impulsive, aggressive, and antisocial behaviour [32,50]. Moreover, cruelty to animals can be found within the diagnostic criteria (DSM-5) [51] for conduct disorder and appears under the criteria for antisocial behaviour within The International Classification of Diseases [52]. Intentional animal cruelty is, however, more common in adolescence and young adulthood, highlighting the need for early prevention. Engagement in animal cruelty during childhood is usually accidental and can therefore be prevented through interventions that promote positive and safe interactions between children and their pets [5]. Exploring the potential impact of children’s engagement in negative interactions on developmental outcomes, such as internalizing and externalizing problems, is therefore important, although has not yet been investigated.

### The Current Study

Evidence into the potential benefits of child–pet interactions for development is increasing; however, there remains limited research investigating the underpinning mechanisms that may contribute to the benefits and risks of HAIs during childhood. The current study, therefore, focused on two key elements of HAIs that are often overlooked within developmental research: (1) the strength of attachment that exists between a child and their pet dog and (2) the types of interactive behaviour that a child engages in with their pet dog, both positive and negative. Such behaviour is important to consider due to the possible mediational effect on the relationship between attachment and developmental outcomes. Pet dogs have been chosen for the focus of this study for several reasons. Children tend to view dogs as attachment figures, display similar attachment features with their dogs to their human attachments, and tend to form strong emotional attachments with pet dogs compared to other pet types [19,20]. This ‘special bond’ that children have with their pet dogs may be explained through the ability to engage in more varied and complex interaction types (e.g., cuddling, playing, even sleeping with) compared to other pet types, through children’s perception that dogs possess similar mental processes to humans, and through the responsiveness of dogs to human emotional states and attachment-related behaviour which facilitates a sense of reciprocity and mutual understanding [22,23,53,54,55]. The overarching aim of the current study was to examine the potential mediating role of caregiver-observed positive and negative child–dog behaviours on the relationship between child-reported child–dog attachment and caregiver-reported child psychopathology and emotion regulation.

## 2. Materials and Methods

### 2.1. Design and Participants

Participants were part of a wider investigation into the emotional and behavioural basis of the child–dog bond that utilized behavioural measures and caregiver and child self-reported measures of pet care practices, pet attachment, and child wellbeing outcomes. The current study focuses on a subset of the data collected and employs a cross-sectional design. Participants were a self-selected, opportunistic sample, recruited through social media channels, public advertising, and school and university newsletters and e-bulletins. Children were recruited via their caregivers. Inclusion criteria were that children had to be aged between 7 and 13 years, have at least one pet dog of any breed and that both children and caregivers were able to give informed consent. Participants with multiple dogs could take part but were asked to focus the questions on the dog that they felt was their child’s ‘favourite dog’, ‘spends the most time with’, ‘or has had the longest’. The mean age of the children was 10 years, 57% were girls and 43% were boys. Mean length of dog ownership was 31 months (SD = 34.53). The full description of caregiver and child characteristics can be viewed in Table 1. The full description of dog characteristics can be viewed in Table 2 and Table 3. The survey had a total of 194 responses, 77 were removed during data cleaning due to missing data or fake responses. The final sample included 117 caregiver reports and 77 fully completed caregiver and child reports.

### 2.2. Procedure

Data were collected between January and August 2021 in the context of the COVID-19 pandemic. The study was conducted according to the guidelines of the Declaration of Helsinki and was approved by the Institutional Ethics Committee of the University of the West of Scotland (approval number: 13308: 11985, date of approval: 10 December 2020). The online survey was hosted on QuestionPro. Caregivers first viewed an information sheet and signed an electronic consent form before being directed to the start of the questionnaire. Caregivers had the option of emailing and/or video calling with the researcher if they wished to ask questions about the study process. Part 1 (caregiver section) of the survey comprised of caregiver report measures, which took approximately 15 mins to complete. Once completed, caregivers viewed a debrief form and could save their answers and close the survey or ask their child to take part in part 2 (child section). Part 2 could be completed later through entering a code for continuing the study. Part 2 began with a child-friendly information sheet and an electronic child consent form before being directed to the questionnaire. It was the caregiver’s responsibility to ensure that their child had read the child version of the information sheet, understood the study and its purpose, and provided informed consent. Children viewed a child-friendly debrief form at the end of the survey.

### 2.3. Measures

#### 2.3.1. Caregiver-Reported Demographic Questions

Caregiver-reported demographic questions included: child’s age, gender, religion, ethnicity, location, if the child had siblings, whether the caregiver was the child’s mother/father/other, whether the child had been ill with COVID-19-related symptoms in the past 2 weeks, whether the family were shielding due to COVID-19 at the time of the study, the level of COVID-19-related restrictions during the time of the study, number of pet dogs in the household and other types of pets, the pet dog’s sex, age, breed, place of dog acquisition, whether the child helped to choose the dog, and whether the child considered the dog to be their own.

#### 2.3.2. Caregiver-Reported Child Behaviour toward the Pet Dog

Caregiver-reported children’s behaviour towards the pet was a measure that was adapted from a previous study [33]. Caregivers were asked “please click the option for how frequently you observe your child to...”, with 29 items relating to positive and negative child behaviour towards the dog, e.g., “speak to the dog”, “hug the dog”, “groom the dog”, “verbally scold the dog”, “throw objects on the dog”, and “lift the dog”. The frequency of each item was rated on a six-point scale from “never” (1) to “very often” (6). Principal component analysis with a varimax rotation was conducted to reduce items into two subscales by fixing the number of extractions to two. Sixteen items were coded into ‘negative behaviours’ such as “yell or scream at during interaction” and “inflict pain accidentally on the dog, e.g., stepping on” (α = 0.99). Thirteen items were coded into ‘positive/benign behaviours’ such as “speak to the dog”, “pet the dog on its body”, and “leave the dog alone when it is resting” (α = 0.99). The average frequency of behaviours for each subscale (positive and negative behaviours) was calculated (Table 4).

#### 2.3.3. Caregiver-Reported Child Psychopathology

Child psychopathology was assessed using the strengths and difficulties questionnaire (SDQ parent-report) [56,57]. This measure contains a total of 25 items rated on a scale of 1–3 (“not true”, “somewhat true”, “certainly true”) based on children’s behaviour and feelings over the past six months. Example items include “considerate of other people’s feelings”, “often has temper tantrums or hot tempers”, “helpful if someone is hurt, upset or feeling ill”, and “has many fears, easily scared”. The measure is comprised of five subscales (emotional symptoms, conduct problems, hyperactivity, peer problems, and prosocial behaviour), and total scores for each subscale can range from 0 to 10. This brief measure is psychometrically sound and is widely used for assessing child mental health problems, with total SDQ scores highly correlating with other measures of psychopathology and clinician-rated child mental disorders [57,58]. Reliability analysis in our sample: total SDQ (α = 0.77), emotional problems (α = 0.82), conduct problems (α = 0.70), hyperactivity (α = 0.65), peer problems (α = 0.63), prosocial (α = 0.70).

#### 2.3.4. Caregiver-Reported Child Emotion Regulation

Children’s emotion regulation abilities were assessed using the Emotion Regulation Checklist (ERC) [59], which is a caregiver report measure that evaluates two dimensions of emotion regulation: negativity and regulation. This measure contains 24 items rated on a 4-point Likert scale from “never” to “almost always”. Example items include “is easily frustrated”, “can say when s/he is feeling sad, angry or mad, fearful or afraid”, “displays flat affect (expression is vacant and inexpressive; child seems emotionally absent)”, “is impulsive”, and “is empathic towards others; shows concern when others are upset or distressed”. Eight items relate to emotion regulation (ER; expression of emotions, empathy, emotional self-awareness), and the total scores for this subscale are calculated (range 8–32), with higher scores indicating greater adaptive regulatory processes. Sixteen items relate to emotional lability/negativity (L/N; lack of flexibility, anger dysregulation, mood lability), and total scores are calculated for this subscale (range 16–64), with higher scores indicating greater emotion dysregulation. This is a widely used measure that demonstrates strong psychometric properties and validity [59] as well as high internal consistency reliability. Reliability analysis in our sample: ER (α = 0.60), L/N (α = 0.65).

#### 2.3.5. Child-Reported Attachment to Dogs

The CENSHARE Pet Attachment Survey (PAS) [60] is a child self-report measure that contains 27 items rated on a 4-point Likert scale from “almost never” to “almost always”. This measure taps into key elements of attachment including relationship maintenance and intimacy. Example items include “you talk to your pet as a friend”, “when you come home, your pet is the first one you greet”, and “you consider your pet to be a member of your family”. To capture additional fundamental aspects of pet attachment ‘pets as a safe-haven’ and pets as a ‘secure-base’, we added three items created by the research team, including “your dog helps you enjoy exploring new places”, “you feel safer when you are with your dog”, and “you feel more confident when you are with your dog”. Negatively worded items are reverse coded and then a total attachment score is calculated (range 30–120). The original measure is suitable to assess attachment to both dogs and cats; however, to provide clarity to children that the questions were about one specific dog, we changed the word “pet” to “dog” across all items. This is a widely used measure for pet attachment that is suitable for our age range and demonstrates strong psychometric properties and validity as well as high internal consistency reliability in our sample; adding the three additional items increased reliability (α = 0.85).

### 2.4. Analysis

A priori power analysis indicated that a minimum sample size of 77 was required to achieve 80% power in detecting a medium effect size based on 3 predictors in the mediation analysis based on an alpha of 0.05. Our parent and child sample sizes were therefore sufficient to detect medium effect sizes. Basic assumptions for mediation analyses were met: all variables were continuous, linear relationships existed between variables, there were no significant outliers when examining scatterplots and studentized residuals (no standardised residuals were ±2.5), there was independence of observations, Durbin–Watson statistic was between 1.5 and 2.5 for all analyses, the data demonstrated homoscedasticity by examining P–P plots, and there was an absence of multicollinearity by examining VIF values (all were below 5). Intercorrelations were carried out using SPSS 25 (IBM SPSS Statistics for Windows, IBM Corp., Armonk, N.Y., USA), followed by parallel mediation analyses using Hayes’ 2013 PROCESS macro for SPSS (V3.5). Completely standardised beta for ab pathways (i.e., indirect effect of X on Y through M) (ab_cs_) can be utilised in mediation analysis to determine the effect size of each indirect effect [61] and Cohen’s effect size standards should be squared in mediation analysis [62,63]. Effect sizes are therefore ab_cs_ = 0.01 (small effect), ab_cs_ = 0.09 (medium effect), and ab_cs_ = 0.25 (large effect). Preliminary analyses found no effect of demographic variables on variables of interest (outlined in Section 2.3.1) and so these did not need to be added as covariates in the mediation analysis. Some demographic variables (e.g., ethnicity, religion, lockdown restrictions, COVID-19 questions, dog acquisition categories, whether children considered the dog to be their own) could not be analysed due to the small numbers in each category.

## 3. Results

### 3.1. Intercorrelations

Descriptive statistics and intercorrelations between variables of interest are displayed in Table 5. Correlations revealed that child–dog attachment significantly positively correlated to positive child–dog behaviours, scores on the prosocial behaviour subscale (SDQ), and emotion regulation. Child–dog attachment significantly negatively correlated with negative child–dog behaviours, emotional problems, conduct problems, hyperactivity, and emotional lability/negativity. Child–dog attachment did not significantly correlate with peer problems. Positive child–dog behaviours significantly positively correlated with scores on the prosocial subscale and scores on emotion regulation. Positive child–dog behaviours significantly negatively correlated with emotional problems and emotional lability/negativity. Negative child–dog behaviours significantly positively correlated with emotional problems, conduct problems, hyperactivity, and emotional lability/negativity. Negative child–dog behaviours significantly negatively correlated with prosocial scores and emotion regulation.

### 3.2. Mediation Analyses

Parallel mediation analyses were carried out to examine the mediating effect of positive child–dog behaviours (M1) and negative child–dog behaviours (M2) on the relationship between child–dog attachment (X) and psychopathology and emotion regulation (Y).

Reversed mediation models were also run to test the directionality of the models by reversing outcomes and mediation variables, therefore testing the mediating role of child psychopathology and emotion regulation (M) as a mediator in the relationship between child–dog attachment (X) and positive and negative child–dog behaviours (Y). These analyses revealed much smaller effect sizes and/or no significant effect, therefore only the non-reversed mediation models are presented in this paper.

#### 3.2.1. Child Psychopathology

Full results from the mediation analysis for child psychopathology are displayed in Table 6.

**Figure 1:**
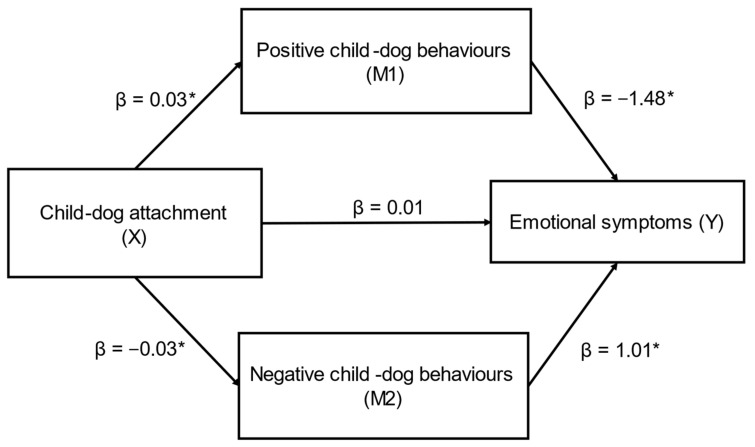
Positive and negative child–dog behaviours as mediators in the relationship between child–dog attachment and emotional symptoms (*n* = 83) (ab_cs_ = −0.33, large effect). Note: * = significant pathway.

Child–dog attachment (X) had a significant indirect effect on caregiver-reported emotional symptoms (SDQ) (Y) through caregiver-reported positive (M1) and negative (M2) child–dog behaviours (ab_cs_ = −0.33, large effect) (Figure 1); this was a complete mediation as the direct effect of X on Y was no longer significant when accounting for M1 and M2. In this model, both positive child–dog behaviours (β = −0.05) and negative child–dog behaviours (β = −0.03) were significant mediators. While positive child–dog behaviours was a marginally stronger mediator, the contrast between positive and negative child–dog behaviours was not significant (β = 0.02, SE = 0.03, CI’s: −0.07, 0.04).

**Figure 2:**
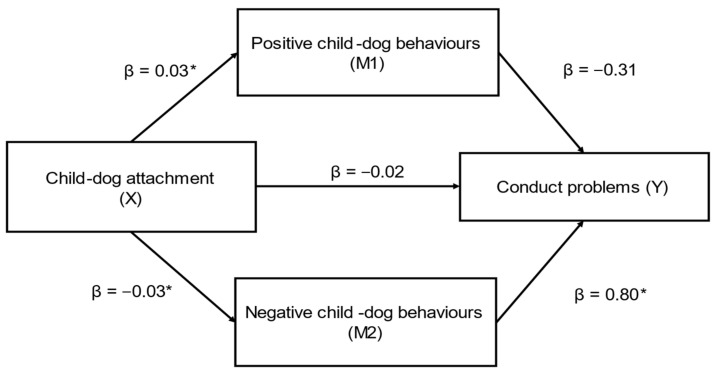
Positive and negative child–dog behaviours as mediators in the relationship between child–dog attachment and caregiver-reported conduct problems (SDQ) (*n* = 83) (ab_cs_ = −0.23, medium effect). Note: * = significant pathway.

Child–dog attachment (X) had a significant indirect effect on caregiver-reported conduct problems (SDQ) (Y) through caregiver-reported positive (M1) and negative (M2) child–dog behaviours (ab_cs_ = −0.23, medium effect) (Figure 2); this was a complete mediation as the direct effect of X on Y was no longer significant when accounting for M1 and M2. In this model, only negative child–dog behaviours was a significant mediator (β = −0.02).

**Figure 3:**
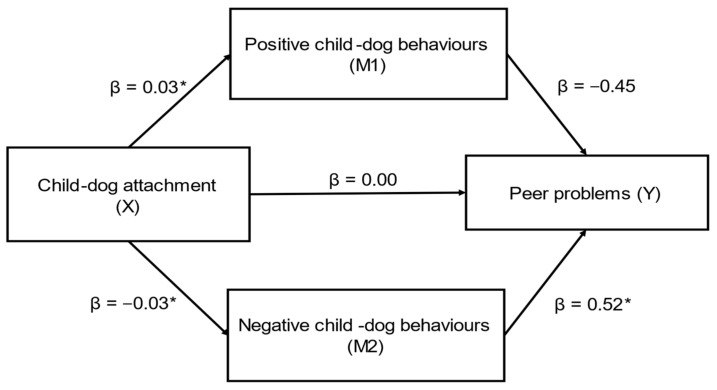
Positive and negative child–dog behaviours as mediators in the relationship between child–dog attachment and peer problems (SDQ) (*n* = 83) (ab_cs_ = −0.27, large effect). Note: * = significant pathway.

Child–dog attachment (X) had a significant indirect effect on caregiver-reported peer problems (SDQ) (Y) through caregiver-reported positive (M1) and negative (M2) child–dog behaviours (ab_cs_ = −0.27, large effect) (Figure 3); this was a complete mediation as the direct effect of X on Y was no longer significant when accounting for M1 and M2. In this model, both positive child–dog behaviours (β = 0.02) and negative child–dog behaviours (β = 0.02) were significant mediators. While positive child–dog behaviours was a marginally stronger mediator, the contrast between positive and negative child–dog behaviours was not significant (β = 0.00, SE = 0.01, CI’s: −0.02, 0.02).

Child–dog attachment (X) did not have a significant indirect effect on caregiver-reported hyperactivity (Y) through caregiver-reported positive (M1) and negative (M2) child–dog behaviours.

**Figure 4:**
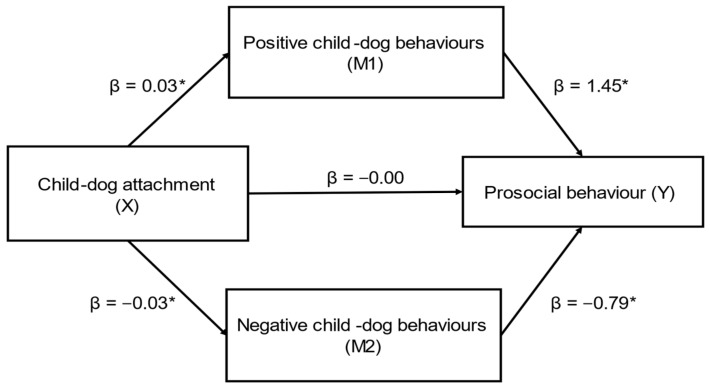
Positive and negative child–dog behaviours as mediators in the relationship between child–dog attachment and prosocial behaviour (SDQ) (*n* = 83) (ab_cs_ = 0.38, large effect). Note: * = significant pathway.

Child–dog attachment (X) had a significant indirect effect on caregiver-reported prosocial behaviour (SDQ) (Y) through caregiver-reported positive (M1) and negative (M2) child–dog behaviours (ab_cs_ = 0.38, large effect) (Figure 4); this was a complete mediation as the direct effect of X on Y was no longer significant when accounting for M1 and M2. In this model, both positive child–dog behaviours (β = 0.05) and negative child–dog behaviours (β = 0.02) were significant mediators. While positive child–dog behaviours was a marginally stronger mediator, the contrast between positive and negative child–dog behaviours was not significant (β = 0.01, SE = 0.04, CI’s: −0.001, 0.03).

#### 3.2.2. Emotion Regulation

**Figure 5:**
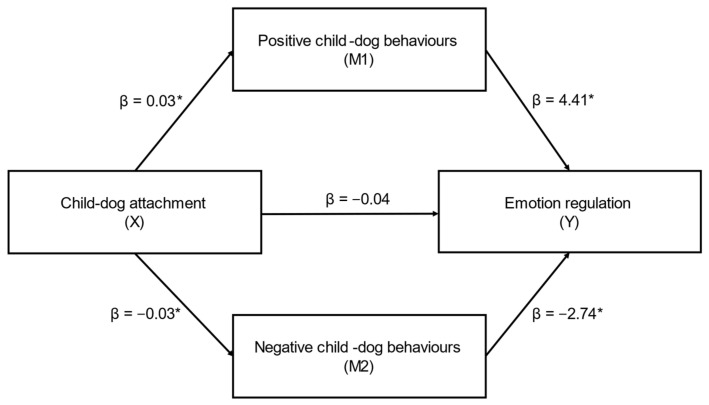
Positive and negative child–dog behaviours as mediators in the relationship between child–dog attachment and emotion regulation (ER) (*n* = 83) (ab_cs_ = 0.45, large effect). Note: * = significant pathway.

Full results from the mediation analysis for emotion regulation are displayed in Table 7. Child–dog attachment (X) had a significant indirect effect on caregiver-reported emotion regulation (ER subscale) (Y) through positive (M1) and negative (M2) child–dog behaviours (ab_cs_ = 0.45, large effect) (Figure 5); this was a complete mediation as the direct effect of X on Y was no longer significant when accounting for M1 and M2. In this model, both positive child–dog behaviours (β = 2.87) and negative child–dog behaviours (β = −1.44) were significant mediators. While positive child–dog behaviours was a marginally stronger mediator, the contrast between positive and negative child–dog behaviours was not significant (β = 0.06, SE = 0.03, CI’s: −0.002, 0.12).

**Figure 6:**
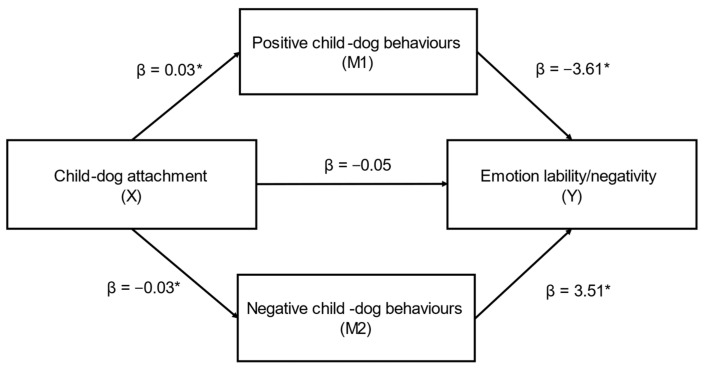
Positive and negative child–dog behaviours as mediators in the relationship between child–dog attachment and emotional lability/negativity (L/N subscale) (*n* = 83) (ab_cs_ = −0.36, large effect). Note: * = significant pathway.

Parallel mediation demonstrated that child–dog attachment (X) had a significant indirect effect on caregiver-reported emotional lability/negativity (EL/N subscale) (Y) through positive (M1) and negative (M2) child–dog behaviours (ab_cs_ = −0.36, large effect) (Figure 6); this was a complete mediation as the direct effect of X on Y was no longer significant when accounting for M1 and M2. In this model, both positive child–dog behaviours (β = −0.12) and negative child–dog behaviours (β = −0.10) were significant mediators. While positive child–dog behaviours was a marginally stronger mediator, the contrast between positive and negative child–dog behaviours was not significant (β = −0.02, SE = 0.06, CI’s: −0.12, 0.11).

## 4. Discussion

The aim of this study was to test the potential mediating role of types of caregiver-observed positive and negative child–dog behaviours on the relationship between child–reported child–dog attachment and caregiver-reported children’s psychopathology and emotion regulation. As predicted, child–dog attachment did have an indirect effect on child psychopathology and emotion regulation abilities through the types of child–dog behaviours observed, although effect sizes were small. These findings therefore provide insight into the potential role that children’s attachment to, and interactions with, pet dogs may play in children’s emotional and behavioural development.

Children who were highly attached to their pet dog engaged in a higher average frequency of positive child–dog behaviours and scored lower on psychopathology and emotion regulation problems. Conversely, children who were less attached to their pet dog engaged in a higher average frequency of negative child–dog behaviours and scored higher on psychopathology and emotion regulation problems. Although caution should be taken when interpreting these findings due to the small effect sizes, these findings are in line with previous theories and existing research [27,28,29,30,31,32] that propose that the strength and quality of the relationship between a child and their pet could impact how that child treats the animal. Stronger attachment can result in a higher likelihood of caring (e.g., feed, groom) and affectionate (e.g., kiss, pet) behaviour, whereas a weaker or lack of attachment can result in fewer positive behaviours and a higher likelihood of negative behaviours (e.g., scream at, attempt to take away food or toys). These negative interactions include both accidental harm (e.g., inflict pain on accidentally) and intentional animal harm (e.g., throw objects at, inflict pain purposefully). These findings therefore point to the important role of the human–animal bond and engagement in positive interactions for companion animal welfare. From a one-health perspective, we can see that it is not only pets that will benefit from the human–animal bond, but that there are also potentially reciprocal human health-related significances.

The current study found that children who were strongly attached to their dog and engaged in more positive and fewer negative interactive behaviours were reported to display fewer emotional symptoms, were more prosocial, had fewer problems with peers, and displayed better emotion regulation abilities. Those who were strongly attached to their dog and engaged in fewer negative behaviours were reported to display fewer conduct problems. These findings support and extend previous investigations, demonstrating an association between pet attachment, prosocial orientations, increased socially positive behaviour, and more humane animal treatment [46,64,65], further supporting the proposed social-facilitating effects of dogs [3,9,46,66]. Overall, these findings corroborate previous theories that human–dog attachment and positive human–dog activities may have beneficial impacts on psychological development [37,53,54] and support the notion that examining dog ownership alone is too simplistic. It is important to explore potentially moderating factors such as attachment and shared activities in human–dog dyads [53,54]. It is important to consider, however, that these variables may only offer ‘one piece of the puzzle’, and the impact of child–pet attachment and interactions on child development may be more complex. There could be an array of additional factors that interplay with such effects (e.g., family and peer influence, early adversity) that should be considered in future research.

When examining human attachment research, there is arguably a strong empirical and theoretical rationale for the directionality of the relationship found between attachment, emotion regulation, and psychopathology. It is well evidenced that attachment problems are a risk-factor for the development of emotional and behavioural disturbances, including internalizing and externalizing problems and difficulties with emotion regulation [44,45,67]. These theories could therefore possibly be extended to human–pet relationships. It could be argued that children with insecure human attachments, who display emotional and behavioural difficulties, may have difficulties forming a secure attachment to a dog, and thus engage in negative interactions. It could also be argued that, when a child lacks a secure human attachment, a secure dog attachment and associated positive interactions are still possible, buffering against the development of psychopathology [4,33]. Assessing a child’s human attachment in addition to pet attachment and examining directionality is therefore an important future research direction. In the current study, although yielding only modest effects, we found that low scores on child–dog attachment predicted psychopathology and emotion regulation difficulties through low engagement in positive interactions. This study did not find a significant mediation effect for caregiver-reported hyperactivity. This finding was unexpected, given the significant positive effect of attachment and positive interactions on emotion regulation, research demonstrating associations between emotion regulation and reduced hyperactivity, and the calming effects of animals for children with hyperactivity disorders [68,69]. The current study did find, however, that, for children who were less attached to their pet dog and engaged in a higher frequency of negative child–dog behaviours, higher scores on conduct problems and emotion regulation problems were reported by their caregivers. Again, although producing only modest effects due to small effect sizes, these findings support the proposed theoretical links between conduct problems, insecure attachment, difficulties in emotion regulation, and compromised animal welfare, and so may have important implications for animal cruelty prevention [32].

The findings further support studies that demonstrate a tendency for aggressive inclinations and antisocial behaviour, including engagement in animal cruelty, when there is a disruption in emotion regulation competencies [32,50], with the current study adding the novel measure of pet attachment. Although strong conclusions cannot be made based on our results, the current findings indicate the possibility of how a child treats a pet animal could be a potential indicator of, or risk factor for, the development of conduct disorder and other co-occurring behavioural disturbances [32,42,50]. Further research on such topics is warranted, especially because appropriate early preventative or intervention strategies may be important in such cases. Future interventions could test the possibility and value of targeting such factors identified in this study (human–pet attachment, positive interactions, emotion regulation skills) to promote the treatment of animals and to improve human developmental outcomes. However, it is important to consider that positive human–pet attachment could act as a buffer against a lack of human–human attachment, thus acting as a protective factor in the development of psychopathology, such as in cases of childhood adversity, where exposure to, and participation in, animal cruelty is more likely to be observed [4,31,33,34]. The current study did not consider exposure to adverse childhood events; therefore, this is a further important research avenue. A strength of this study is that it focused on the general population, whereas previous studies investigating HAIs and psychopathology have tended to focus on clinical or forensic populations or sub-groups of children displaying emotional and/or behavioural disturbances [32,50]. However, this meant that most of the children in our study displayed ceiling-level attachment to their pet dogs, displayed high engagement in positive interactions and low engagement in negative behaviours and displayed low rates of internalizing and externalizing problems (see Table 5 for all means). Nevertheless, our study indicates that not all child–dog relationships are positive and that some children in the general population are engaging in negative interactions and may be having difficulties in forming an attachment to their pet dog. Given the significance for animal welfare and possibly child development, further attention is warranted.

### Limitations and Future Directions

There are limitations of this study that need to be considered when interpreting the findings. This study was carried out during the COVID-19 pandemic, at a time when many participants were spending an unusual amount of time at home. This increased time spent at home increased the number of opportunities for a child to both engage in and bond with their pet dog, as well as an increased chance of caregivers observing a wider range of child–dog behaviours that perhaps may have been missed or even may not have existed pre-pandemic, thus potentially exaggerating the findings. There is also a reliance on caregivers accurately reporting on their child’s behaviour, and so, as with all survey data, there is a risk of desirability bias that could possibly be mitigated in future studies through triangulation, such as the inclusion of behavioural observations and interviews. Due to data being collected during the pandemic, difficulties arose with reaching and recruiting caregivers and their children in the research study despite several online recruitment strategies. This resulted in a relatively small sample size, limiting the generalizability of the findings. The sample was, however, large enough to detect a medium effect size in the mediation analyses based on a priori power analysis.

As with most human–animal research, those who did choose to take part may have represented a sample where there is high interest in and strong attachment to pet dogs, thus increasing bias and limiting the diversity of our sample. Future studies could consider different recruitment strategies and advertisement wording and imagery used to encourage a more representative sample. This lack of diversity also meant that some demographic variables could not be statistically analysed due to the small numbers of participants in the categories. There may be other confounding variables that were not accounted for in the current study such as dog–child behaviours, dog temperament, and dog breed [70]. Due to the diversity of dog breeds and small numbers within each breed, inter-breed differences could not be examined in this study, yet children may feel more attached to and may be more inclined to engage in certain activities with, particular breeds [70]. The current study also examined the impact of pet dogs on child development cross-sectionally during a critical window in childhood where there is a rise in pet acquisition [9]. Follow-on studies could improve upon this study by examining these variables longitudinally across developmental periods and/or at important additional times of transitions in a young person’s development, such as during adolescence and emerging adulthood, especially because pet attachment and engagement with pets may peak and fall at different developmental time points [70].

Finally, this study included a measure that assessed the total strength of emotional attachment to a dog, rather than assessing child–dog attachment types. Presently, there are no existing child-friendly measures that assess different pet attachment types, yet it is well known that attachments vary in emotional involvement and commitment and that human–animal attachments, similar to human–human attachments, can also feature anxieties, insecurities, and avoidance (as observed in adult samples [21,24,26,27]). Developing, validating, and making a widely and freely available new child-friendly measure that taps into differing dimensions of pet attachment (anxiety and avoidance) would provide the opportunity to develop a more nuanced understanding of the complexities and individual differences in child–pet attachments and its subsequent impact on developmental outcomes. Further research is also required to examine how and when children form attachments to pets and how a secure attachment to a pet could be promoted.

## 5. Conclusions

Human–animal research is currently limited and over-simplistic due to overlooking the importance of individual differences in the attachment that exists (or does not exist) between a child and their pet dog, and the varied interactions that children engage in with that pet dog, on developmental outcomes. The present study is the first to examine the mediating role of the types of interactions that occur between children and their pet dogs on the relationship between emotional attachment to dogs and child psychopathology and emotion regulation. Although caution should be taken due to modest effect sizes, this study indicates the possibility that a weaker (or lack of) attachment and engagement in negative child–dog behaviours could pose a possible risk for emotional and behavioural problems in childhood and could increase the risk for both intentional and unintentional animal cruelty. Further investigation with a larger sample size to increase statistical power is therefore warranted and should take into account additional factors that may interplay with such effects such as adverse childhood experiences. There may be important implications of such work for the development and evaluation of interventions and prevention strategies that aim to promote positive and safe human–pet interactions, which may subsequently impact a child’s psychological development.

## Figures and Tables

**Table 1:** Caregiver and child characteristics (*n*).

**Child’s Ethnic Group**	Category	White British	White Traveller	Other White	Mixed/Multiple Ethnic Groups—White and Asian	Other *
	*n*	97	3	9	4	2
**Child age**	Category	7–8 years	9–10 years	11–12 years	13 years	
	*n*	23	45	39	10	
**Location**	Category	United Kingdom	United States	Canada	Brazil	Australia
	*n*	80	28	4	3	2
**Family system**	Category	Older sibling/s	Younger sibling/s	Twin sibling	No siblings	
	*n*	54	40	7	16	
**Child’s religion**	Category	No religion	Christian (all denominations)	Hindu	Muslim	Other/prefer not to say
	*n*	57	55	2	1	2
**Caregiver taking part**	Category	Mother	Father	Other		
	*n*	91	19	7		
**COVID-19 restrictions at time of study**	Category	Full lockdown	High number of restrictions	Moderate number of restrictions	Limited restrictions	No restrictions
	*n*	57	22	18	9	11
**Had child been ill with COVID-19 related symptoms in past 2 weeks?**	Category	No	Yes–confirmed	Yes–not confirmed		
	*n*	111	5	1		
**Shielding at time of study due to COVID-19?**	Category	No	No but did so within past month	Yes		
	*n*	103	5	9		

Note: * Other Mixed/Multiple ethnic background (*n* = 1), Asian/Asian British–Chinese (*n* = 1).

**Table 2:** Pet dog characteristics (*n*).

**Place of Dog Acquisition**	Category	Breeder	Rescue from Home Country	Rescue Abroad	Family Friend	Other
	*n*	67	12	3	28	8
**Dog sex**	Category	Male	Male neutered (castrated)	Female	Female neutered (spayed)	
	*n*	34	26	22	35	
**No. of total dogs in household**	Category	1	2–3	4–5	6 or more	
	*n*	88	27	2	0	
**No. of total pets in household**	Category	1	2–3	4–5	6 or more	
	*n*	58	47	9	3	
**Did the child help to choose the dog?**	Category	Yes	No	Dog already present at childbirth		
	*n*	90	20	7		
**Does child consider dog to be their own?**	Category	Yes	No	Unsure		
	*n*	82	28	7		

**Table 3:** Pet dog breeds (*n*).

Breed	*n*	Breed	*n*	Breed	*n*
Cocker Spaniel Cavapoo/Cockapoo/Cockashipoo	18	Labradoodle/Goldendoodle	6	Jack Russell	3
Labrador	12	Husky/Malamute	8	Poodle/Miniature Poodle	3
Golden Retriever	10	English Springer Spaniel/Sprocker/Sprollie	4	Bull Terrier (Staffordshire Bullterrier cross, English Bull Terrier, American Pitbull Terrier)	3
Mixed/Unknown	9	Pug/Pug cross	4	English Bulldog/British Bulldog/French Bulldog	3
Border Collie/Collie Cross/Rough Collie/Welsh Collie	8	German Shepherd	3	Border Terrier	2
Beagle/Beagle cross	2	Chihuahua/Pinscher	2	Whippet	2
Bichon Frise	2	Other *	13		

* Flat-coated Retriever, American Foxhound, German Shorthaired Pointer, Rhodesian Ridgeback, Shih Tzu, Lhasa Apso, West Highland White Terrier, Dalmatian, Dobermann, Setter, Australian Kelpie, Miniature Schnauzer, Lab/Spaniel Cross (all *n* = 1).

**Table 4:** Descriptive statistics for caregiver-reported positive and negative child–dog behaviours. Minimum 1, maximum 6.

Negative Child–Dog Behaviours	M	SD	Positive Child–Dog Behaviours	M	SD
Pull on body parts of the dog, e.g., tail, ears	1.59	1.11	Feed the dog	4.03	1.44
Inflict pain deliberately on the dog, e.g., hitting	1.40	1.11	Groom the dog	3.15	1.64
Attempt to take away the dog food or bowl	1.58	1.28	Hug the dog	5.30	1.13
Throw objects on the dog	1.51	1.14	Pet the dog on its body	5.51	0.94
Inflict pain accidentally on the dog, e.g., stepping on	1.80	1.08	Reach for the dog	4.87	1.42
Sit, lie, or ride on the dog	1.88	1.46	Pet the dog on its head	5.30	1.15
Restrain the dog by its collar	2.22	1.33	Approach or follow the dog	4.66	1.41
Attempt to take dog toys/chews from the dog	2.29	1.57	Kiss the dog	4.46	1.61
Yell or scream during interaction	2.06	1.35	Leave the dog alone when it is resting	3.85	1.43
Attempt to pet the dog when it is eating or drinking	2.08	1.59	Lead the dog on a leash	3.83	1.56
Dress the dog	1.90	1.40	Lay down near to the dog when it is resting	4.06	1.45
Take child toys from the dog	2.81	1.63	Request obedience from the dog/give commands	4.31	1.45
Verbally scold the dog	2.06	1.11	Speak to the dog	5.43	1.11
Involve the dog in child play, e.g., doctor game	2.34	1.49			
Wake the dog when it is sleeping	2.83	1.51	Average frequency of positive child–dog behaviours	4.52	0.68
Lift the dog	2.55	1.63	Average frequency of negative child–dog behaviours	2.06	0.92

**Table 5:** Descriptive statistics and intercorrelations among main study variables.

	M	SD	1	2	3	4	5	6	7	8	9	10
**1** Attachment	95.55	10.89	1	0.48 **	−0.24 *	−0.25 *	−0.33 **	−0.16	−0.24 *	0.30 **	0.31 **	−0.44 **
**2** Positive child–dog behaviours	4.52	0.68		1	0.07	−0.26 **	−0.10	−0.16	−0.03	0.38 **	0.42 **	−0.34 **
**3** Negative child–dog behaviours	2.06	0.92			1	0.28 **	0.21 *	0.19	0.20 *	−0.27 **	−0.32 **	0.49 **
**4** Emotional symptoms	2.40	2.53				1	0.47 **	0.38 **	0.34 **	−0.38 **	−0.63 **	0.72 **
**5** Conduct problems	1.66	1.46					1	0.40 **	0.34 **	−0.25 **	−0.36 **	0.44 **
**6** Peer problems	2.90	1.26						1	0.18	−0.03	−0.33 **	0.32 **
**7** Hyperactivity	2.72	1.83							1	−0.05	−0.17	0.38 **
**8** Prosocial behaviour	7.92	1.90								1	0.62 **	−0.55 **
**9** Emotion regulation	35.47	4.78									1	−0.68 **
**10** Emotional lability/negativity	23.62	6.39										1

Note: Effect sizes are: small, r = 0.1; medium, r = 0.3; large, r = 0.5 (Cohen, 1992). ** Correlation is significant at the 0.01 level (2-tailed). * Correlation is significant at the 0.05 level (2-tailed).

**Table 6:** Parallel mediation analysis examining indirect effects of child–dog attachment (X) on caregiver-reported child psychopathology (Strengths and Difficulties Questionnaire, SDQ) (Y), via caregiver-reported positive child–dog behaviours (M1) and negative child–dog behaviours (M2).

	Emotional Symptoms			Conduct Problems			Peer Problems			Hyperactivity			Prosocial Behaviours		
	β	SE	95% CI	β	SE	95% CI	β	SE	95% CI	β	SE	95% CI	β	SE	95% CI
Completely standardised indirect effect beta values of X on Y (ab_cs_) (total)	−0.33 *	0.10	−0.53, −0.16	−0.23 *	0.10	−0.43, −0.05	−0.27 *	0.10	−0.48, −0.08	−0.05	0.09	−0.02, 0.01	0.38 *	0.09	0.21, 0.56
Direct effect of M1 on Y	−1.48 *	0.48	−2.42, −0.53	−0.31	0.27	−0.86, 0.23	−0.45	0.23	−0.91, 0.02	0.08	0.38	−0.66, 0.83	1.45 *	0.35	0.75, 2.16
Direct effect of M2 on Y	1.01 *	0.29	0.43, 1.59	0.80 *	0.17	0.47, 1.14	0.52 *	0.14	0.24, 0.81	0.40	0.23	−0.06, 0.86	−0.79 *	0.22	−1.22, −0.36
Direct effect of X on Y	0.01	0.03	−0.05, 0.07	−0.02	0.02	−0.06, 0.01	0.00	0.02	−0.03, 0.03	−0.03	0.02	−0.08, 0.02	−0.00	0.02	−0.05, 0.04
Indirect effect of X on Y via M1	−0.05 *	0.02	−0.09, −0.01	−0.01	0.01	−0.03, 0.01	−0.02 *	0.01	−0.03, −0.00	0.00	0.01	−0.02, 0.03	0.05 *	0.01	0.02, 0.08
Indirect effect of X on Y via M2	−0.03 *	0.02	−0.07, −0.01	−0.02 *	0.01	−0.04, −0.01	−0.02*	0.01	−0.04, −0.00	−0.01	0.01	−0.03, 0.00	0.02 *	0.01	0.01, 0.04
Unstandardised total indirect effect of X on Y via M1 and M2	−0.08*	0.02	−0.13, −0.04	−0.03 *	0.02	−0.07, −0.01	−0.03 *	0.01	−0.06, −0.01	−0.01	0.02	−0.04, 0.02	0.07 *	0.02	0.04, 0.11

Notes: * Significant pathway (*p* < 0.05). Effect sizes: ab_cs_ = 0.01 (small effect), ab_cs_ = 0.09 (medium effect), and ab_cs_ = 0.25 (large effect). M1 = positive child–dog behaviours. M2 = negative child–dog behaviours.

**Table 7:** Parallel mediation analysis examining indirect effects of child–dog attachment (X) on caregiver-reported emotion regulation (Y), via caregiver-reported positive child–dog behaviours (M1), and negative child–dog behaviours (M2).

	Emotion Regulation (ER)			Emotional Lability/Negativity (L/N)		
	β	SE	95% CI (LL, UL)	β	SE	95% CI (LL, UL)
Completely standardised indirect effect beta values of X on Y (ab_cs_) (total)	0.450 *	0.10	0.26, 0.66	−0.27 *	0.08	−0.45, −0.13
Direct effect of M1 on Y	4.41 *	0.78	2.85, 5.96	−2.08 *	0.93	−3.93, −0.22
Direct effect of M2 on Y	−2.74 *	0.48	−3.69, −1.78	2.22 *	0.57	1.08, 3.35
Direct effect of X on Y	−0.04	0.04	−0.11, 0.03	−0.07	0.06	−0.19, 0.05
Indirect effect of X on Y via M1	2.87 *	0.55	1.79, 3.96	−0.12 *	0.04	−0.20, −0.05
Indirect effect of X on Y via M2	−1.44 *	0.33	−2.11, −0.78	−0.10 *	0.04	−0.19, −0.03
Unstandardised total indirect effect of X on Y via M1 and M2	0.14 *	0.03	0.08, 0.20	−0.22 *	0.05	−0.33, −0.13

Note: * Significant pathway (*p* < 0.05). Effect sizes: ab_cs_ = 0.01 (small effect), ab_cs_ = 0.09 (medium effect), and ab_cs_ = 0.25 (large effect). M1 = positive child–dog behaviours. M2 = negative child–dog behaviours.

## Data Availability

The data presented in this study are available on request.
